# JAK2 V617F Mutation Leading to Portal Vein Thrombosis in a Young Patient: A Case Report

**DOI:** 10.7759/cureus.76547

**Published:** 2024-12-28

**Authors:** Chandrashekar Patil, Venkata Ramya Ananthu, Mohd Abdul Haq Junaid, Polneni Lavanya, Safiya Jabeen

**Affiliations:** 1 Radiodiagnosis, Malla Reddy Medical College for Women, Hyderabad, IND

**Keywords:** cytoreductive therapy, jak 2 mutation, myeloproliferative neoplasm, portal vein thrombosis (pvt), portal vein thrombosis [pvt], thrombocythemia

## Abstract

Myeloproliferative neoplasms (MPNs) are clonal hematopoietic stem cell disorders commonly characterized by excessive production of blood cell lineages. The JAK2 V617F mutation plays a crucial role in the pathogenesis of these conditions, often leading to thrombotic complications. Here, we present the case of a 21-year-old man who presented with acute abdominal pain and was found to have portal vein thrombosis with splenomegaly. Imaging studies confirmed chronic portal vein thrombosis with extensive collateral circulation. Laboratory evaluation revealed an elevated platelet count, and genetic testing confirmed the presence of the JAK2 V617F mutation, suggestive of essential thrombocythemia. The patient was managed with anticoagulation (apixaban) and cytoreductive therapy (hydroxyurea). This case emphasizes the importance of considering underlying MPNs in young patients presenting with unusual thrombotic events, and highlights the significance of early diagnosis and targeted treatment in improving patient outcomes.

## Introduction

Myeloproliferative neoplasms (MPNs) are disorders of clonal hematopoietic stem cells characterized by the overproduction of blood cell lineages. The presence of a JAK2 (Janus kinase 2) gene mutation is a significant factor in the pathogenesis of these disorders [1]. The JAK2 V617F mutation is found in most patients with essential thrombocythemia (ET), polycythemia vera (PV), and primary myelofibrosis (PMF) [2]. This mutation results in constitutive activation of the JAK-STAT signalling pathway, leading to increased proliferation of hematopoietic cells independently of external cytokine signals [3].

The discovery of the JAK2 V617F mutation has transformed the understanding, diagnosis, and treatment of MPNs, providing a molecular explanation for previously heterogeneous clinical presentations [1,4]. Although the mutation is not exclusive to any specific MPN, its identification has become an essential diagnostic criterion, aiding in classification and risk assessment. Patients with the JAK2 mutation often present with varied clinical features, such as erythrocytosis, leukocytosis, or thrombocytosis, and have an increased risk of complications like thrombosis and disease progression [5,6].

## Case presentation

A 21-year-old man presented to the emergency department with complaints of squeezing abdominal pain for the past three days. The patient reported that the pain began abruptly and progressively worsened over time, significantly affecting his ability to carry out daily activities. He denied any history of trauma, recent travel, or alcohol consumption, and there were no prior similar episodes. The pain was constant, progressively worsening, and localized to the upper abdomen without radiation. There were no symptoms of vomiting, fever, or jaundice. On physical examination, the patient appeared uncomfortable, with tenderness in the left upper quadrant, but without guarding or rebound tenderness. His vital signs were stable, with no signs of hypotension or tachycardia. The abdominal examination was notable for splenomegaly, while there were no signs of hepatomegaly or ascites. An abdominal ultrasound was performed, revealing splenomegaly and a decreased caliber of the main portal vein with multiple periportal collaterals, suggestive of chronic portal hypertension. Based on these findings, a contrast-enhanced computed tomography (CECT) scan of the abdomen was recommended for further evaluation of the portal venous system.

**Figure 1:**
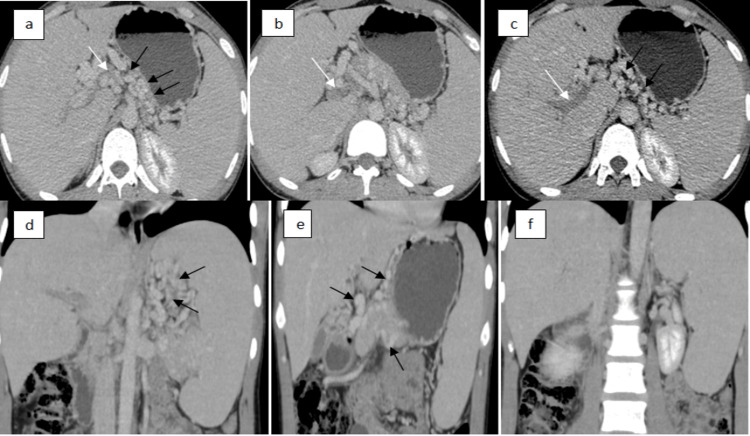
Contrast-enhanced computed tomography scan of the abdomen (a and b) Axial venous phase images showing absent enhancement of the main portal vein (white arrows), suggestive of complete thrombosis; (c) axial venous phase image showing complete thrombosis of the right branch of the portal vein (arrow), with multiple portal collaterals seen in (a) to (c) (black arrows); (d and e) coronal venous phase images depicting multiple portal collaterals around the portal vein, lesser sac, and along the lesser and greater curvature of the stomach (black arrows); (f) significant hepatosplenomegaly

CECT revealed significant findings, including splenomegaly, absent contrast opacification in the main portal vein and right portal vein, and severe narrowing of the left portal vein branch with absent contrast opacification (Figures 1a-1c). Multiple portal collaterals (Figures 1a-1f) were noted at the hilum, peripancreatic, intrapancreatic, perigastric, perisplenic, and along the gallbladder and fundic wall. Portal collaterals were also noted in the anterior abdominal wall mesentery. These findings were consistent with chronic portal vein thrombosis with resultant development of extensive collateral circulation.

Initial laboratory investigations, including a hemogram, revealed an elevated platelet count of 470,000/µL, while hemoglobin concentration, hematocrit levels, and white blood cell counts were within normal ranges. The elevated platelet count raised suspicion of an underlying myeloproliferative disorder.

The subsequent sequence analysis of genomic DNA identified the JAK2 V617F mutation, leading to the diagnosis of an MPN, likely essential thrombocythemia. Given the findings of portal vein thrombosis, the patient was started on anticoagulation therapy with apixaban (tablet) to prevent further thrombotic events, and hydroxyurea (capsule) to manage the elevated platelet count and reduce the risk of thrombosis.

## Discussion

This case highlights the rare occurrence of portal vein thrombosis in a young patient with an underlying JAK2-positive myeloproliferative neoplasm. Portal vein thrombosis is an uncommon but serious complication of MPNs, which can lead to significant sequelae such as portal hypertension, splenomegaly, and the development of extensive collateral circulation. The identification of the JAK2 V617F mutation was instrumental in determining the underlying cause of the patient's hypercoagulable state, which allowed for the appropriate therapeutic interventions.

Patients with MPNs, especially those with JAK2 mutations, are at increased risk of thrombotic events due to several mechanisms, including hyperviscosity, increased platelet activation, and endothelial dysfunction [4]. These factors collectively contribute to a pro-thrombotic state, which can manifest as venous thrombosis, including portal vein thrombosis, and other complications. The hyperviscosity is primarily due to the increased red cell mass or elevated platelet count, while the activated platelets and endothelial dysfunction promote an inflammatory and pro-coagulant environment.

In this case, the presentation of portal vein thrombosis, particularly in a young patient, underscores the importance of considering underlying hematologic disorders such as MPNs in the differential diagnosis. Portal vein thrombosis in young individuals is rare and typically warrants investigation into pro-thrombotic conditions. The elevated platelet count, in conjunction with imaging findings and the genetic confirmation of the JAK2 V617F mutation, pointed towards an underlying essential thrombocythemia as the likely etiology.

The treatment strategy for this patient included the use of hydroxyurea, a cytoreductive agent aimed at controlling the elevated platelet count and thereby reducing the risk of further thrombotic complications. Hydroxyurea acts by suppressing hematopoietic stem cell proliferation, which helps in normalizing platelet counts and mitigating hyperviscosity. Apixaban, a direct oral anticoagulant, was initiated to address the thrombotic risk associated with the portal vein thrombosis and to prevent further clot formation. The combination of cytoreductive therapy and anticoagulation is considered the standard approach in managing MPNs with thrombotic complications.

The importance of early recognition and management of MPNs cannot be overemphasized, especially in cases presenting with unusual complications such as portal vein thrombosis. Timely intervention with cytoreductive therapy and anticoagulation can significantly improve outcomes by preventing progression to more severe sequelae, including variceal bleeding due to portal hypertension or transformation to a more aggressive disease state like myelofibrosis or acute leukemia [5].

This case also emphasizes the evolving role of genetic testing in the diagnosis and management of hematologic disorders. The identification of the JAK2 V617F mutation not only confirmed the diagnosis but also allowed for risk stratification and guided therapeutic decisions. The presence of the mutation indicates a higher risk of thrombotic events, thereby necessitating vigilant monitoring and a proactive treatment approach.

Other than overproduction of blood cells, the JAK2 mutation can also lead to bone marrow fibrosis, splenomegaly and increased clotting risk potentially causing deep vein thrombosis, pulmonary embolism, heart attack, and stroke. In some cases, these disorders can evolve into acute myeloid leukemia in later stages. The JAK2 mutation can also lead to thrombosis of other vessels such as splenic vein, superior mesenteric vein, hepatic veins and cerebral venous sinuses. Rao and Grosel reported a similar case of portal vein thrombosis in a 59-year-old patient with JAK2 V167F mutation. The patient presented to the emergency department with abdominal pain, nausea, weight loss and poor appetite. CT was done that demonstrated decreased attenuation in the main portal vein extending from the main portal vein into the porta hepatis. On further investigations, the patient was diagnosed to have JAK2 V167F mutation. The patient was discharged on enoxaparin [7].

## Conclusions

The presence of the JAK2 V617F mutation has significant implications for diagnosis, prognosis, and management. This case report highlights the importance of considering an underlying myeloproliferative neoplasm in young patients presenting with thromboembolic events, as seen in our patient with portal vein thrombosis and elevated platelet counts. Early diagnosis and timely initiation of appropriate treatment, including cytoreductive therapy and anticoagulation, are essential to reduce the risk of thrombotic complications in patients with MPNs.
